# The long journey towards a shift to rail in the European long-distance passenger transport market

**DOI:** 10.1038/s44333-025-00025-9

**Published:** 2025-02-03

**Authors:** Oded Cats

**Affiliations:** Department of Transport & Planning, Faculty of Civil Engineering and Geosciences, Delft University of Technology, Stevinweg 1, 2628 CN Delft, The Netherlands

**Keywords:** Sustainability, Geography

## Abstract

The European long-distance passenger transport market is rapidly changing. There is a striking discrepancy between the relevance of long-distance travel for emission reduction goals and the lack of knowledge to support its design, planning and policy making. A conceptual representation of this market is provided, and four key scientific challenges are identified and discussed along with a brief review of the state-of-the-art, related knowledge gaps and a corresponding research agenda.

## Current state-of-affairs

In the past century, long-distance passenger travel has become increasingly common. While bringing many advantages by means of enhanced mobility and connectivity, it also comes at a cost due to the externalities generated, such as the depletion of finite natural resources, noise pollution and its contribution to climate change. In 2019, the transport sector produced 29% of all GHG emissions in the European Union^1^. It is estimated that while making up only 2.5% of all trips in Europe, long-distance trips—defined as longer than 100 kilometres in the respective study by Petersen et al.^2^—generate more than half of all passenger-kilometres travelled. Expectations made during the COVID pandemic that air travel, the dominant travel mode in the long-distance travel market, will not recover to its pre-pandemic levels or that long-distance travel patterns will fundamentally shift have already been largely debunked with air traffic levels nearly fully recovered to their pre-pandemic level^3^.

This perspective article is concerned with the prospects of a shift to rail in the long-distance travel market. Consequently, we focus in the following on trips which can reasonably take place by trains. Trains, and in particular High-Speed Rail (HSR), are often considered a promising alternative for short-haul flights and long-distance car travel while inducing fewer environmental disadvantages for the range of 200 to 1500 kilometres^4^. This is therefore the range considered in the remaining of this article. Below this range car is the dominant travel alternative and above this range flights are the most competitive mode of travel. In particular, given the large differences in CO2 emission rates between air and HSR^5^, stimulating a shift towards the latter is critical. The European Court of Auditors^6^ stated however that the current state amounts to “*a patchwork of poorly connected national HSR networks*”. Hence, despite the seemingly favourable circumstances (i.e. high density urban centres within hundreds of kilometres distance, high wealth levels, developed national rail networks, supranational organisations), a European HSR-network is yet to be realised. Remarkably, the number of cross-border trains in Europe has hardly increased between 2001 and 2019. More so, the number of cities connected by cross-border traffic decreased significantly during the same period^7^. This grim state of affairs can be partially explained by planning authorities adopting a national perspective on the evaluation of cross-border connections thereby undermining network effects and yielding an overall sub-optimal outcome^8^.

Governmental bodies at both national and European levels are calling for an urgent need to take actions aimed at reducing the externalities associated with air travel, in particular environmental ones. This has galvanized commitments for large-scale investments in developing a European HSR-network^9^. This is accompanied by a growing public and political debate concerning measures meant to discourage flying as well as around promoting alternatives for air travel. Despite the abovementioned commitments, current developments are unlikely to yield a significant modal shift. For the EU to meet its climate strategy in transport an investment of €25 billion per year is required, most of it for rail infrastructure, given the key role of modal shift and the insufficient progress made insofar^10^. Moreover, infrastructure investment alone is insufficient in achieving a better international train service. The main bottlenecks in developing an attractive international rail transport system pertain, next to infrastructure also to each of the other three system layers—traffic system, transport services and mobility services^11^. Related recent innovations include the rapid growth of night train services, open-access rail operators, attempts to improve integrated air-rail connections, and the pioneering introduction of Mobility as a Service (MaaS) platforms in the long-distance context. In addition, an array of supply- and demand-management strategies aimed at fundamentally reshaping how we travel are put forward such as introducing airport capacity caps, short-haul flights restrictions and the allocation of mobility credits.

## The long-distance passenger transport service market

Long-distance transport services are heavily regulated, require large capital investments and relevant expertise, and are operating in a highly constrained infrastructure environment resulting in high entry barriers and a number of strong market actors. At the same time, long-distance transport services are market-driven and prices are highly dynamic due to the prevalence of revenue management practices.

**Fig. 1:**
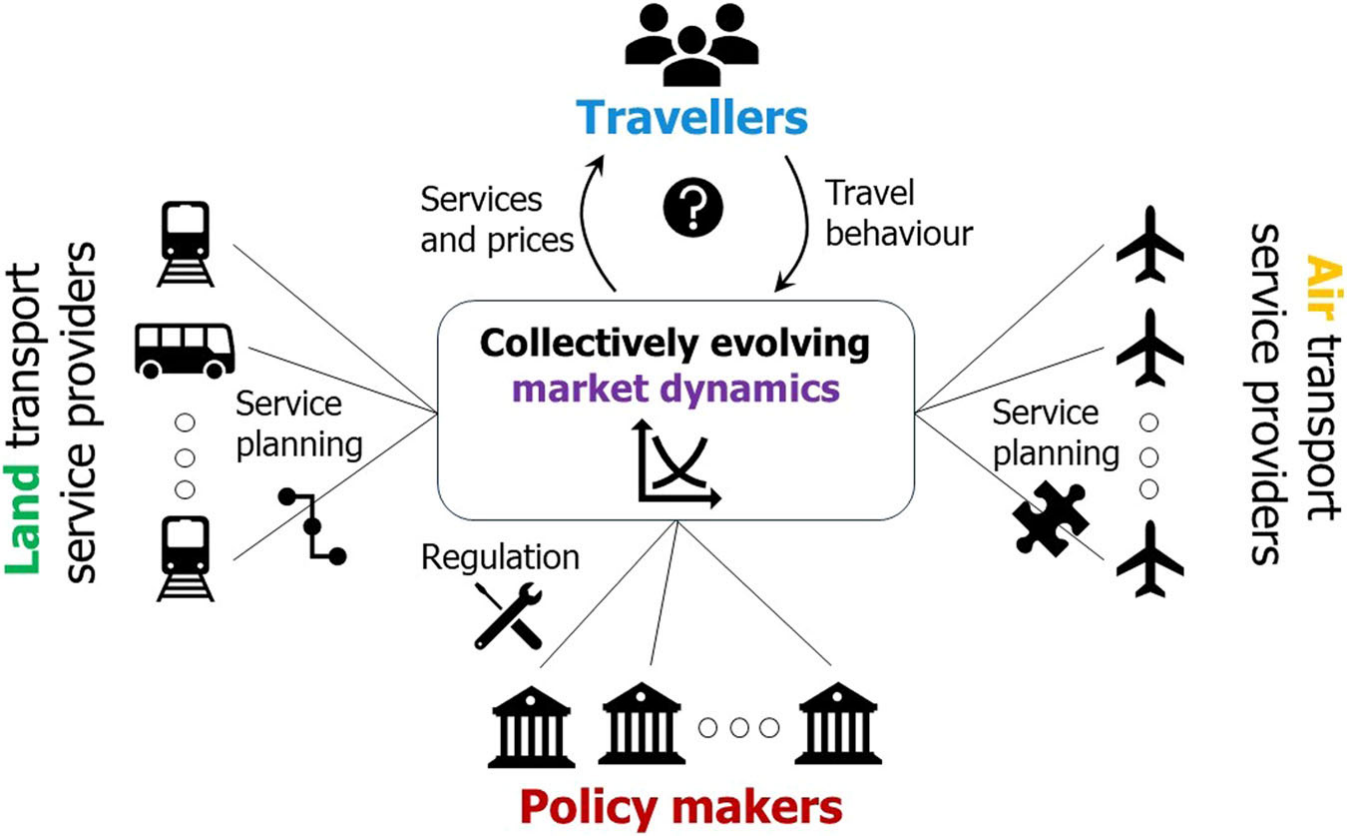
Key actors and related processes in the context of long-distance passenger transport service market. The long-distance travel market is conceptualized as the place where on the one hand supply offered by air and land-bound—both rail and bus (coaches)—transport service providers and on the other hand demand—individual transport service users (i.e. travellers)—interact. The outcomes of the long-distance transport market depend on the interactions between service providers and travelers subject to the interventions made by policy makers.

Figure 1 depicts the key system actors—providers (supply), users (demand) and policy makers (regulation)—in the long-distance passenger transport service market and their interactions. In the following each of these set of players is briefly discussed.

Transport service providers are responsible for developing their network of connections, service lines and associated timetables, as well as designing and operating the resulting vehicle and crew scheduling. These companies set prices, sell tickets (directly or via mediators), manage resources, buy access from the infrastructure managers, and form alliances with other actors.

Travellers make decisions based on their needs, preferences and constraints. These decisions are not limited to the mode and itinerary choices involved in choosing between passenger transport services—bus, rail and air—and a rented, or if applicable, a privately owned vehicle. Rather, due to the dominant role of costs in non-mandatory and non-habitual travel decisions and the large fluctuations in price levels, ticket prices impact not only travellers’ mode-route choice decisions but also higher-level travel dimensions such as destination choice and whether to travel altogether (corresponding to the trip distribution and trip generation steps in the classic 4-step transport demand modelling framework). Travel cost is therefore not only an important choice determinant but also a limiting variable in determining the choice-set composition, thereby making certain alternatives exclusive. Other barriers to making a meaningful choice between travel alternatives include the ability to access and process relevant information (e.g. related to familiarity, technology or language barriers), time availability constraints (e.g. having to return home within a certain time window may not permit taking a given mode) or legal status (e.g. having to show travel documents at border controls).

The outcomes of the long-distance transport market depend on the interactions between service providers and travelers subject to the interventions made by policy makers. the latter are particularly complex given the cross-border nature of long-distance travel in the European context where multi-level governance arrangement entail that different authorities have spatially varying jurisdictions ranging from the local and regional to the national, supra-national and in the case of air travel even global scale. Consequently, questions of international geopolitics play a role for example in relation to climate goals, protectionism and restrictions set on cross-border traffic of both people and vehicles. Future developments in relation to, potentially differentiated, (dis)integration of European countries will impact not only the degree of integration of European long-distance transport markets but also the demand for cross-border transport. Governance bodies may intervene in the market in the form of setting requirements for service coordination, rules for the interactions between transport service providers and the infrastructure managers, and legislation (e.g. passenger rights, short-haul flight restrictions).

While authorities set policies and regulations, each service provider designs its own network of services based on the current market state (rather than by a central planner) thereby resulting in a network-of-networks. The latter emerges as an outcome of interactions between a large number of actors following a combination of top-down and bottom-up principles and reflects the steady-state order, i.e. equilibrium conditions, which prevail for a certain development phase and market conditions. In contrast to bus (coach) transport which offers a low-cost alternative and an agile network of connections, rail depends on large-scale infrastructure investments and as such its development greatly depends on the outcomes of political process and the success of governance and coordination efforts.

## A brief review of the state-of-the-art

The relation between supply and demand in the long-distance transport market and the resulting system performance is arguably fundamentally different from those of other transport markets. While air and rail service providers compete for travellers, at the same time they can also offer complementary services exhibiting both within- and cross-network effects. Evaluating and forecasting how the long-distance transport market will develop involves four key challenges pertaining to (i) travellers’ preferences, (ii) network assignment and design, (iii) market dynamics, and (iv) policy design and evaluation. In the following I provide a brief review of the state-of-the-art for each of which.

### Travel demand and behaviour

Past studies on disaggregate long-distance demand characteristics have focused on specific aspects of air travel choices using either stated preferences or revealed preferences data. This includes airport choice from a set of nearby airports, airline preferences, itinerary choice, and airport access mode choice^12–15^. Hierarchical Information Integration (HII) approach was adopted to elicit travellers’ preferences in relation to complex constructs such as perceived safety^16^ and comfort^17^. While these studies provide a solid empirical understanding of the key determinants of specific choice elements, these choice estimates are commonly stand-alone models, hindering their integration in large-scale transport demand models, as pointed out by Kristoffersson & Berglund^18^ in their review of modelling connecting access/egress trips to long-distance travel terminals. Notable exceptions of mode choice studies leveraging revealed preferences data include the works of Ortúzar & Simonetti^19^ who estimated mixed stated and revealed preference models for the choice between air and a fictious HSR connection in Chile and of Román et al.^20^ who estimated a mode choice model based on revealed preferences data for the Madrid-Barcelona corridor and found large heterogeneity in value of time estimates. Moreover, there is evidence that HSR users have a distinctive socio-economic profile^21^.

Several studies examined the role of environmental awareness in inducing modal shift in long-distance travel, primarily from air to rail. There is however growing evidence that points to a persistent “vast attitude behavior gap” between pro-environmental attitudes and the choices travellers make^22,23^. Moreover, a growing body of research concludes that attitudes and knowledge/awareness play a limited role in impacting pro-environmental attitudes and behaviour^24,25^. Instead, results from a meta-analysis indicate that the most impactful “interventions designed to change social-structural determinants” are “policies that increase access to a particular behaviour”^24^.

### Network assignment and design

Network assignment—the distribution of travel demand over an underlying graph representing transport connections—plays a key role in any transport planning undertaking and is used as part of project appraisal procedures so as to assess generalized travel costs, level-of-service and capacity needs. There have been some isolated efforts to develop assignment models for either HSR or airline networks. Cascetta & Coppola^26^ analyzed the extent to which frequency- and schedule-based models—the two alternative principles used in public transport assignment models—could be used for the analysis of HSR services. Conversely, Hsiao & Hansen^27^ are amongst the few that developed an assignment model for air passenger flows and Birolini et al.^28^ estimated an itinerary choice model and demonstrated its application for assigning demand to the network of outbound trips from Italian airports.

In designing passenger transport systems service providers aim at maximizing the connectivity offered by their network by enabling interchanges between service lines. While challenged by the emergence of the point-to-point business strategy undertaken by low-cost airlines, national airlines strive at consolidating flows through strengthening their hub-and-spoke network. Related work on the analysis of airline networks from complex networks lens examined the underlying mechanisms which may lead to the emergence of this structure^29^ as well as its robustness for the European rail and air networks and the combination thereof^30^. Numerical experiments and sparse empirical observations suggest that competition in hub-and-spoke networks can generate negative network externalities and even reduce total social surplus^31^. Top-down approaches for solving either air of rail network planning problems have been commonly formulated and solved by means of optimisation models. For example, Birolini et al.^32^ formulated an airline network planning model which considered demand-supply interdependencies by embedding a supply-dependent travel flow function into the optimisation problem and Liu et al.^33^ formulated a profit-oriented objective for the Chinese HSR network, albeit where passenger demand is considered constant and exogenously determined. Grolle et al.^34^ developed a customized service network design and frequency setting problem for the European high-speed rail network where demand for rail services is modelled endogenously (i.e. ridership depends on level-of-service).

### Market dynamics

The long-distance transport market is characterised by a plurality of commercial parties—ranging from legacy state-backed to low-cost airlines and from national railway companies to open-access rail companies—operating in a competitive yet highly regulated environment. The airline industry is long known for its revenue management practices and the work of Littlewood^35^ pioneered with a mathematical model for the airline revenue management problem which has been followed by many advancements and became an industry standard. In the past decade, behavioural models regarding travellers’ purchasing choices have been used for estimating air price elasticities^36^ and incorporated into air^37^ and rail revenue management^38^. Adler et al.^39^ formulated an optimization problem for obtaining the prices, vehicle capacity and service frequency set by airlines and HSR operators with travellers choosing one or the other.

Notably, service providers are not only competitors but also potential collaborators, giving rise to coopetition practices such as forming airline alliances^40^ and offering integrated air-rail products^41^. While the substitutability and complementarity relations between HSR and air transport have been subject to ex-ante assessments^42–44^, the relation between supply availability and the demand and prices that prevail in the market are yet to be established. This is critical in order to forecast the implications of different market settings on passenger flows, ticket prices and market shares.

### Policy design and evaluation

Measures for mitigating the environmental impacts of long-distance travel can be classified into supply- and demand-management instruments. The two most commonly discussed supply-management measures include the introduction of capacity caps for airport slots and short-haul flight bans. Assessments of the impacts of the latter concluded that its environmental consequences are very limited^45^ and do not offset travel time losses^46^, while others pointed to the need to increase rail capacity in order to absorb the anticipated demand shift from air^47^.

Demand management strategies aim at setting the right incentives so as to stimulate more sustainable travel behaviour by the internalization of the emission costs. Taxation is the most commonly applied instrument (e.g. airport taxes attached to flight tickets). For example, Belgium considers to introduce additional taxes for purchasing tickets for flights within certain spatial limits and members of the Dutch parliament proposed charging extra taxes from passengers who fly to the Netherlands in order to transfer rather than as a final destination. Büchs and Mattioli^48^ analysed the distributional effects of alternative forms of taxing air travel and concluded that they are all distributionally neutral or progressive. An alternative demand management scheme involves the introduction of tradeable mobility credits (TMC). It can be compared to existing schemes which have been proven to be successful, such as the EU emission trading system (ETS), fishing quotas, and airport slot allocations. In contrast to these schemes, TMC are distributed and consumed by individuals and not on a firm or industry level. While in the case of taxation schemes the price is fixed and total emissions are not directly limited but rather the outcome of travelers’ choices, for TMC this is the other way around, i.e. the total emissions are fixed by design whereas the price is the outcome of travelers’ choices^49^. Results reported by Tanner et al.^50^ suggest that the introduction of TMCs for leisure travel in Europe will result in a reduction of 20% in travel demand and in the share of air among the remaining trips decreasing from 50 to 42% whereas the market share of rail will increase from 23 to 26%. Notwithstanding, lack of knowledge on the underlying dynamics of the long-distance transport market hampers forecasting the impacts of future scenarios and interventions.

## Knowledge gaps and a research agenda

The scientific challenges in the long-distance travel context are diverse and profound. In the following I briefly outline the related knowledge gaps and a related a research agenda comprising of recommendations for research directions corresponding to the four abovementioned research areas. While the focus of this perspective article is on the European context and market, the research agenda outlined below is relevant also for other world regions as well as for connections between Europe and those other world regions.

### Travel demand and behaviour

There is a striking lack of knowledge on route choice decisions in the context of long-distance train travel (e.g. valuations of reliability and transfers) as well as in the context of integrated air and rail services. Past studies of air itinerary choices—whether based on stated or revelated preferences—have been limited to the consideration of alternative itineraries. Consequently, the inter-relation between modal choice in the long-distance context and higher-order choices such as trip destination and the choice of travelling altogether—which are key behavioural responses to changes in market and policy conditions—is largely unknown. Birolini et al.^28^ provide indeed evidence based on the analysis of aggregate data that air transport supply significantly affects not only trip destination choice but also trip making. Furthermore, additional empirical underpinning is needed regarding the factors that make people choose between direct and indirect flights as well as access and egress modes. It is important to recognize that the tourism and hospitality sectors (e.g. hotels, convention and event centres) are important players in determining the origin-destination demand for long-distance passenger transport market for both business and leisure purposes. This calls for more research at the intersection of tourism and transport demand studies.

### Network assignment and design

Despite the urgent need for methods and tools to support the design of integrated long-distance services and policies, there is no model which allows for multi-layer and multi-modal long-distance network assignment, i.e. where passengers can combine and transfer between a series of legs of different modes within a single path (itinerary). Unlike individual vehicle (e.g. car, bike) traffic, the modelling of passenger service networks such as air, bus and rail require in addition to a graph representation of the underlying infrastructure also a graph representation of the service layer which is superimposed on the respective infrastructure (also known as L- and P-spaces, see Luo et al.^51^). Furthermore, in both air and HSR contexts^52,53^ there is evidence to suggest that there are significant differences amongst travellers which can be well-described by (probabilistically) partitioning the population into market segments. A multi-class assignment model is therefore required to super-impose different traveller flows in a multi-layer multi-modal network. Network design studies have hitherto assumed a single designer, either an agency or an operator or a single objective function accounting for both perspectives. It remains thus unknown how the network-of-networks—which is the outcome of interactions between network design decisions made by a large number of service providers and the collective outcome of individual travellers’ decisions—emerges and evolves. Future research may therefore develop game theoretical or co-evolutionary models for reproducing the network design process. Such models will enable capturing the co-existence of point-to-point and hub-and-spoke network structure strategies undertaken by different market players.

### Market dynamics

The role of strategic behaviour by market players and coopetition (competition-collaboration) practices in determining market states and outcomes remains largely unknown. This hinders the analysis of substituteability and complementarity relations between alternative transport service providers. Key scientific challenges pertain to the design of price setting and coalition formation mechanisms in multi-modal markets with strategic behaviour, coopetition and network effects which are the outcome of bottom-up, decentralised, profit maximization decision-making by numerous players. Service providers are likely to adopt strategic behaviour in for example extending their network giving rise to notions such as critical mass and phase transitions where the agglomeration effects generate positive network effects. The different underlying principles of flow agglomeration and cost functions for rail, bus and air are expected to yield distinctive topologies and pricing strategies as well as exercise path-dependencies.

### Policy design and evaluation

Arguably, the impacts of both supply- and demand-management policies remain largely unknown because of the lack of empirical knowledge on the extent of behavioural responses as well as market adaptations made by a plurality of long-distance transport service providers. Furthermore, potential re-bound effects—such as assigning the newly available slots to more lucrative long-haul flights and the re-distribution of passenger flows towards feeding services connecting to airports located outside the flight ban perimeter—may result with counter-intuitive and even counter-productive outcomes. For example, there is substantial empirical evidence from China, Japan and Europe pointing to the impact of HSR on the redistribution of demand over airports and its potential stimulation of international air traffic, resulting in an unclear overall impact on emissions^54–56^. Lack of knowledge on the underlying dynamics of the long-distance transport market hampers forecasting the impacts of future scenarios and interventions. Past attempts to assess the consequences of alternative scenarios neglected both demand and supply responses to policy interventions^57,58^. Future research should assess the implications of different policy trajectories in terms of emissions generated, induced or reduced demand levels, modal split, and accessibility and distributional effects. The latter pertaining to both regional and socio-economic disparities. Furthermore, in order to support robust decision making and identify potential no-regret policies, it is essential to evaluate the effectiveness of alternative policy measures under different scenarios in terms of travel trends, political and technological developments (e.g. reduced globalism in tourism, increased/decreased power and budget for supranational authorities, acceleration and cost-reduction of electric aircrafts deployment).

## Conclusion

To conclude, in addressing these challenges, a combination of behavioural, network and market models are needed to assess the consequences of various scenarios (e.g. demand levels and distribution, technological and economic developments) and decisions taken by service providers and authorities (e.g. revenue management, decarbonisation goals, HSR infrastructure). The shift to rail is not inevitable. Arguably, it is rather improbable if not supported by empirical evidence and robust models to devise policies and evaluate developments related to long-distance travel. In the absence of which, we risk devising poor and expensive or even harmful policy instruments as well as performing infrastructure appraisals based on unsolid grounds.

## Data Availability

No datasets were generated or analysed during the current study.
